# A Fault-Tolerant Data Fusion Method of MEMS Redundant Gyro System Based on Weighted Distributed Kalman Filtering

**DOI:** 10.3390/mi10050278

**Published:** 2019-04-26

**Authors:** Binhan Du, Zhiyong Shi, Jinlong Song, Huaiguang Wang, Lanyi Han

**Affiliations:** Department of vehicle and electronics, Army Engineering University of PLA, Shijiazhuang 050000, China; dubinhan@163.com (B.D.); sjzsong_jl@163.com (J.S.); ins10208@163.com (H.W.); hly_hanlanyi@163.com (L.H.)

**Keywords:** multi-sensor, data fusion, distributed Kalman filtering, fault tolerance, parity space

## Abstract

The application of the Micro Electro-mechanical System (MEMS) inertial measurement unit has become a new research hotspot in the field of inertial navigation. In order to solve the problems of the poor accuracy and stability of MEMS sensors, the redundant design is an effective method under the restriction of current technology. The redundant data processing is the most important part in the MEMS redundant inertial navigation system, which includes the processing of abnormal data and the fusion estimation of redundant data. A developed quality index of the MEMS gyro measurement data is designed by the parity vector and the covariance matrix of the distributed Kalman filtering. The weight coefficients of gyros are calculated according to this index. The fault-tolerant fusion estimation of the redundant data is realized through the framework of the distributed Kalman filtering. Simulation experiments are conducted to test the performance of the new method with different types of anomalies.

## 1. Introduction

The micro electro-mechanical system inertial measurement unit (MEMS IMU) has the advantages of being low cost, small volume and light weight, but the low precision and poor stability are still problems that cannot be ignored. Many aircrafts, cars, and satellites are beginning to use MEMS sensors. Among them, a large portion of MEMS accelerators have already met tactical and navigation grade requirements. However, only a few MEMS gyros can reach the level of the tactical grade [1], and these tactical grade gyros are too expensive for common application. To improve the performance of MEMS IMU, a series of studies have been conducted to promote the MEMS manufacturing technology. On the other hand, the improvement can also be achieved through reasonable designs and algorithms. Under the restriction of the current manufacturing technology, the redundant design is the most effective approach to improve the accuracy and stability. The redundant measurement signals can be fused to produce a more accurate signal. Besides, the reliability of the system can also be greatly improved. Even for the tactical or navigation grade sensors, the redundant configuration can improve the performance substantially [2]. 

The most necessary part of the redundant inertial navigation system (RINS) is the management of the redundant data, which includes two aspects: Data fusion estimation and anomaly data processing. The anomaly data is comprised of two categories to be processed: The outliers are processed by the outlier eliminating algorithms or the outlier rejecting filtering [3,4]; and the faults are detected and isolated by the fault detection and isolation (FDI) technology [5,6,7]. The methods of data fusion include the virtual gyroscope technology [8], distributed Kalman filtering [9,10], federated Kalman filtering [11], least squares (LS) method [12,13] and various improved algorithms based on them. In a complete system, after obtaining the measurement data at each sampling moment, the measurement data is examined for the presence of outliers, and then the faults are detected through FDI methods. If there is any fault, the faulty device will be isolated and the redundant system will be reconstructed. Finally, the measurement data from normal devices is fused and estimated by the data fusion algorithms.

However, in practice experiments, several problems are found in the current strategy. First, the outliers in the measurement data, especially the outlier patches, are easily misdiagnosed as the faults, which will lead to an increase in the false alarm rate. The outliers will cause the detection function values to exceed over the detection threshold, but there is no failure or damage in the sensors with outliers. Such misdiagnosis may induce a well-behaved sensor to be deactivated. Second, when the outlier eliminating methods and FDI methods are operating simultaneously, the practical performance may degrade, because the calculation burden will increase and the results of different methods will interfere with each other. For example, the outlier eliminating methods regard every anomaly point as an outlier. Since the deviation of anomaly points caused by sensor faults will be diminished after the outlier eliminating processing, the original statistical characteristics of the faults will change. As a result, the probability of correct detection (PCD) and the probability of correct isolation (PCI) will decline. Third, it is difficult to detect incipient faults, which have not exceeded the detection threshold, but such faults will also cause impact on the accuracy of the system. Finally, most of the current data fusion algorithms are based on the assumption of the unbiased estimation. But the outliers and faults will undermine the unbiasedness, and it will cause the accuracy of the fusion estimation decrease.

In order to solve the problems above, this paper designs a fault-tolerant fusion algorithm for the redundant MEMS gyro systems. The proposed method provides a real-time monitoring of the redundant inertial information with the covariance matrix and fault detection and isolation function. The measurement data quality index is designed as the evidence of the weighted fusion estimation algorithm. Based on the structure of the distributed Kalman filtering, a fault and outlier tolerant fusion method is presented. In this method, the outliers and faults are processed simultaneously to avoid the first two problems mentioned above. The measurement data quality index is sensitive to each variation of the local measurement signal, and the missed detection for the incipient faults and minor faults is improved. The index not only considers the random noise but also reflects the unbiasedness of measurement signals, so the last problem mentioned above is solved. The experiments demonstrate that the proposed method can improve the accuracy and stability of MEMS IMU. The redundant data management process is simplified, and the computing burden of the system is reduced.

This paper is organized as follows. The weighted distributed Kalman filtering is briefly reviewed in Section 2. In Section 3, the singular value decomposition (SVD) based FDI method is reviewed, and the improved methods are presented. In Section 4, the disadvantages of the traditional strategy are analyzed. In Section 5, the improved index of the measurement data quality is proposed based on the fault detection and isolation function, and the developed fault-tolerant fusion method are proposed in this section. The simulation experiments are conducted to test the performance of the new method, and the results are compared with other methods in Section 6. Finally, the conclusions are given in Section 7.

## 2. Multi-Sensor Optimal Information Fusion Kalman Filtering Weighted by Scalar

In this section, a distributed Kalman filtering [9,10] is reviewed briefly. This algorithm is a weighted estimation under the requirement of the linear minimum variance. Compared with the centralized fusion estimation, it is globally suboptimal. But it is optimal in the linear unbiased minimum variance criterion under the structure of the weighting local estimation. Considering a multi-sensor system with n sensors, $x_{0}$ is the real state to be estimated. $x_{i}$ (i = 1, 2,…, n) is the measurement data of the i-th sensor. $\sigma_{i}^{2}$ is the variance matrix of noise in i-th sensor. The fusion method can be given by the equations as follows.

(1) $${\hat{x}}_{0}=\sum_{i=1}^{n}w_{i}{\hat{x}}_{i}$$

(2) $$W=\frac{P_{tr}^{-1}e}{e^{T}P_{tr}^{-1}e}$$

Among the equations above, ${\hat{x}}_{i}$ is the unbiased estimate of the i-th sensor, and ${\tilde{x}}_{i}$ is the estimation error. ${\hat{x}}_{0}$ is the fusion estimation value of the system. The optimal weight coefficient vector is given by $W={\left[w_{1},w_{2},\cdots ,w_{n}\right]}^{T}$. The matrix $P_{tr}$ and vector e can be defined as:

(3) $$P_{tr}=\left[\begin{array}{c} \begin{array}{ccc} \mathrm{tr}(P_{11}) & \cdots & \mathrm{tr}(P_{1n}) \end{array}\\ \begin{array}{ccc} \vdots & & \vdots \end{array}\\ \begin{array}{ccc} \mathrm{tr}(P_{n1}) & \cdots & \mathrm{tr}(P_{nn}) \end{array} \end{array}\right],\;e=\left[\begin{array}{c} 1\\ \vdots \\ 1 \end{array}\right]$$

$P_{ij}$ is the covariance matrix of the estimation error ${\tilde{x}}_{i}={\hat{x}}_{i}-x_{i}$ and ${\tilde{x}}_{j}={\hat{x}}_{j}-x_{j}$, and the tr(·) indicates the trace of matrix. The estimation error covariance matrix of the optimal weighted fusion algorithm is calculated by:

(4) $$P_{0}=\sum_{i=1}^{n}\sum_{j=1}^{n}w_{i}w_{j}P_{ij}$$

The relation proved in [10] is given as follows:

(5) $$\mathrm{tr}(P_{0})\leq \mathrm{tr}(P_{ii})\begin{array}{cc} & i=1,\cdots ,n \end{array}$$

**Figure 1:**
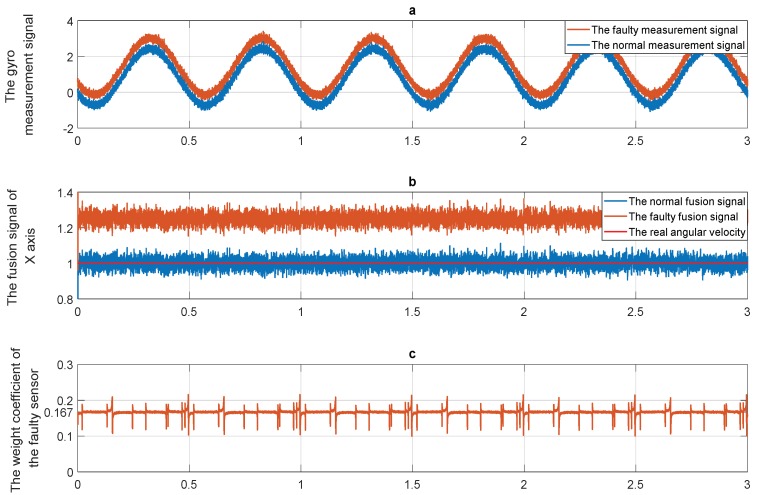
The simulation experiment in the two situations: (**a**) The gyro measurement signals in normal and faulty situations; (**b**) the fused signals of the X-axis in normal and faulty situations; (**c**) the weight coefficient of the faulty gyro.

To sum up, the weight coefficient of each sensor in this method is dependent on the traces of covariance matrixes. A necessary premise is that each local estimator is unbiased. Therefore, if any anomaly occurs in the inertial sensors, the unbiasedness will be destroyed. As a result, the final estimation accuracy will decrease. To explain this point, a simulation experiment is presented in Figure 1. The 6-gyro-dodecahedron redundant system is adopted in the simulation, a 6σ constant drift fault is injected into the measurement signal of the fourth gyro. This experiment is briefly described here, and the detailed description is presented in Section 6. Figure 1a shows the normal signal and the faulty signal. The redundant gyro signals are fused through the method introduced above, and the fusion signals in the normal situation and faulty situation are compared as Figure 1b shows. The weight coefficients of the faulty gyro are shown in Figure 1c. Table 1 exhibits the detailed information.

From Figure 1b and Table 1, compared with the normal case, the noise variance of the faulty fusion signal increases by 0.218 times, but the bias (constant drift) of the faulty signal is about 500 million times larger than the normal case. In the simulation, the fourth gyro has a conspicuous fault as Figure 1a shows. Ideally, the weight coefficients of the different gyro are equal in the normal situation (which is 1/6 in this experiment). When the gyro has faults, the corresponding coefficient is supposed to decrease. But from Figure 1c, the weight coefficinet of the faulty gyro still remains at around 1/6 (the mutation is caused by the outliers). Therefore, the conclusion can be drawn that the traditional fusion method is only sensitive to the noise but not sensitive to the bias. This is the reason why the nosie variance of the faulty case only increases a little, but the bias increases significantly.

## 3. The Fault Detection and Isolation Method

### 3.1. The Singular Value Decomposition Based Method and its Improvement

The parity space method is a diagnosis method based on the analytic redundancy. Its basic idea is to construct a parity matrix, which is based on the hardware redundancy or analytical redundancy equations of the system. Then, the parity (consistency) of the system mathematical model (analytic redundancy relationship) is checked with the actual observation. The methods need to find the parity equation decoupled from the faults, and the parity vector and the fault detection function are constructed based on the parity equation. With the detection threshold determined by the hypothesis testing theory, the faults can be detected once the detection function values exceed the threshold. In this paper, the SVD based method [5] is reviewed, and the relations between detection functions and data anomalies are deduced. This relation is the basis to analyze anomalies.

In normal working, the observation equation of a redundant system composed of n gyroscopes can be expressed as follows:

(6) Z=Hx+ε

In the equation, Z is the measurement data vector of *n*-dimensional; $H_{n\times 3}$ is the geometric configuration matrix with n rows and three columns; x is a three-dimensional vector of angular rate state; ε is the n-dimensional white Gaussian noise vector, which statistical characteristics meet:

(7) $$E[\varepsilon ]=0,E[\varepsilon \varepsilon^{T}]=\sigma^{2}I_{n}$$

When the anomalies appear, the observation equation can be expressed as:

(8) Z=Hx+b+ε

In Equation (8), b is the anomaly vector. To detect anomalies, in [5], an SVD-based method was presented, and a detailed derivation and verification were conducted. The first step is to decompose the geometric configuration matrix H through the singular value decomposition.

(9) $$H=U_{H}\left(\begin{array}{c} S_{H}\\ 0 \end{array}\right)L_{H}^{T}$$

In Equation (9), the matrix $S_{H}$ is a diagonal matrix. The decoupling matrix V is constructed form the matrix $U_{H}$ as follows.

(10) $$U_{H}=[U_{1}\vdots U_{2}]$$

(11) $$V=U_{2}U_{2}^{T}$$

where $U_{1}\in R^{n\times 3}$ and $U_{2}\in R^{n\times (n-3)}$.

The parity vector p is calculated with the matrix V and the measurement vector Z as follows.

(12) p=VZ

In [5], the fault detection function and isolation function are given as follows:

(13) $$F_{D}=p^{T}p$$

(14) $$F_{{}_{I}}^{i}=p^{T}V_{i}$$

where the $V_{i}$ is the fault reference vector, which refers to the i-th column of the matrix V, and the $F_{{}_{I}}^{i}$ corresponds the i-th sensor. The decision rule to detect fault is given as follows.

(15) $$\{\begin{array}{c} H_{0}:F_{D}<T_{D}\\ H_{1}:F_{D}>T_{D} \end{array}$$

In [5], it is suggested that the $F_{D}$ obeys the $\chi^{2}$ distribution, so the threshold $T_{D}$ can be determined with a given false alarm probability α. If $H_{0}$, then the system can be considered fault-free; if $H_{1}$, then proceed to the isolation step. The fault isolation function of each sensor is calculated out. Next, the largest isolation function $F_{I}^{k}$ is found, and the k-th sensor is isolated.

### 3.2. The Improvement of the SVD Based Method

**Figure 2:**
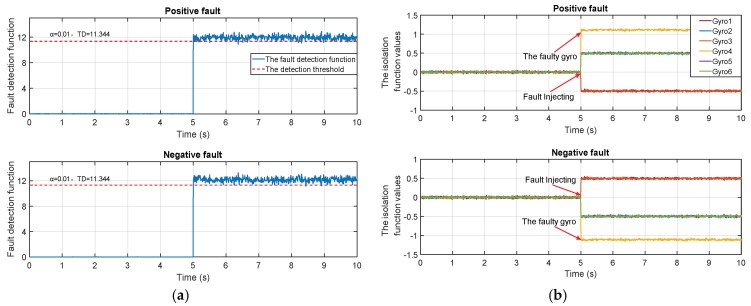
The fault detection and isolation function in two cases: (**a**) The detection functions of the positive fault and negative fault; (**b**) the isolation functions of the positive fault and negative fault.

The method above has the following disadvantages, which is analysed and proved in reference [14]:

(1) The detection function *F*_D_ has the probability to disobey the $\chi^{2}$ distribution, because the $\chi^{2}$ distribution requires that each variable is independent. The matrix V is singular, so elements of the vector p=VZ are not independent. As a result, the threshold determined by $\chi^{2}$ distribution is inappropriate.

(2) The isolation function $F_{{}_{I}}^{i}$ given in Equation (14) may cause the inequality of isolation probability among the sensors. The isolation function is designed to indicate the similarity between the parity vector and the fault reference vector, and the sensor with the highest similarity is most likely to have faults. From Equation (14), the values of the isolation function are subjected to the modules of $V_{i}$, but there is no guarantee that the modules of each fault reference vector $V_{i}$ are equal. The similarity between the two vectors is supposed to only depend on the projection or the angular between them. In other words, the modules of the fault reference vectors are not supposed to influence the isolation function. A more appropriate method is to use the angular between the parity vector and the fault reference vector ***V**_i_*. For example, consider a simple situation: $V_{1}={\left[0,1\right]}^{T}$, $V_{2}={\left[4,0\right]}^{T}$ and $p={\left[1,\sqrt{3}\right]}^{T}$. The isolation function values are calculated with Equation (14), and there are $F_{{}_{I}}^{1}=\sqrt{3}$ and $F_{{}_{I}}^{2}=4$. So the conclusion from the original method regards the second senor as faulty. However, the angular between $V_{1}$ and p is 30°, and the angular between $V_{2}$ and p is 60°. It is obvious that the first sensor is most likely to have faults, and the original method makes a wrong decision.

(3) The negative faults can cause the method to produce an incorrect result. For example, a group of gyro measurement signals are simulated based on the 6-gyros-dodecahedron system. A positive step fault and a negative step fault are injected in the fourth gyro in turn. The signal, detection function and isolation function are shown in Figure 2. Analyzing the figure, the detection function is able to detect the faults once the faults are injected. However, the isolation strategy isolates the sensors, which corresponds to the largest isolation function value. However, the negative fault will bring down the isolation function values of the faulty sensor. Therefore, the isolation function fails to isolate negative faults.

To solve the problems above, the method is improved as follows. For the first problem, the decoupling matrix V is redefined and the parity vector is standardized as follows.

(16) $$V=U_{2}^{T}$$

(17) p=VZ/σ

Since this improvement, the $p^{T}p$ obeys *χ*^2^(*n* − 3) distribution, and the threshold can be determined with a given false alarm rate α.

To solve the last two problems, the isolation function is redefined as:

(18) $$F_{{}_{I}}^{i}={\left(\frac{p^{T}V_{i}}{\left|p\right|\left|V_{i}\right|}\right)}^{2}=\frac{{\left(p^{T}V_{i}\right)}^{2}}{V_{i}^{T}V_{i}p^{T}p}$$

The improved isolation function indicates the angular between the parity vector p and the i-th fault reference vector $V_{i}$, and the effect of the module of $V_{i}$ is eliminated. So the imparity caused by fault reference vectors will not influence the isolation results. The negative-fault problem can also be solved through the squaring operation in Equation (18).

## 4. The Disadvantages of Current Fault-Tolerant Fusion Strategy

In the section above, the data fusion method and the SVD based FDI method are introduced. In this section, the disadvantages of the current fault-tolerant fusion strategy are analysed, and the simulation experiments are conducted to prove our viewpoints.

The traditional strategy of the fault-tolerant fusion strategies need to eliminate the outliers first; then the outlier-eliminated signals are diagnosed through the FDI method, and the faulty sensors are isolated; finally, the fusion estimation results are calculated with the measurement signals from anomaly-free sensors.

The first flaw is the problem of the false alarm and the missed detection. For the false alarm, the outlier is a common phenomenon in the measurement system. The definition of an outlier is an observation (or subset of observations) that appears to be inconsistent with the remainder of that set of data. The outliers arise in the inertial measurement data because of the instrument error, natural deviations, or changes in behaviour of systems, which usually appear as the self-recovery and transient mutations [3]. This kind of anomalies can make the fault detection function exceed the threshold like the faults. The traditional strategy regards the system as faulty once the detection function exceeds the threshold, so the sensors with outliers are easily misdiagnosed as faulty devices. However, such sensors have no damage or failure.

**Figure 3:**
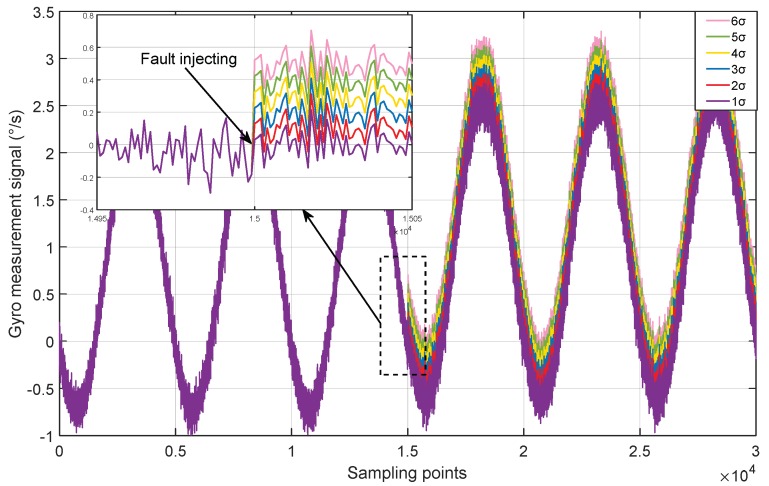
The gyro measurement signals with various constant drift faults.

**Figure 4:**
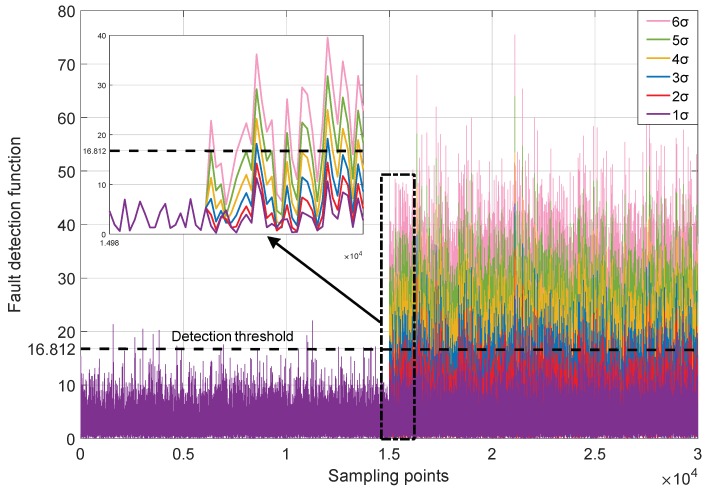
The fault detection functions of different gyro measurement signal.

To solve the problem, most detection methods diagnose the system as faulty, when the anomaly detection function values exceed the detection threshold at a plurality of consecutive points, or exceed the threshold at a large part of points within a certain period of time. This strategy will bring the missed detection problem. For example, we use six MEMS gyros with the variance of $\sigma \approx 0.105\;({}^{\circ }/s)$ to build a RINS, and the redundant structure adopts the regular dodecahedron. The constant drift fault in varying deterioration degrees is injected into the normal measurement signals and the FDI performance is tested through the improved SVD based method. The measurement signals are simply marked as “1σ”, “2σ”, …, “6σ”, which means the nσ constant drift fault is injected into the original signal. The details are shown in Figure 3 and Figure 4 and Table 2.

It can be seen from Table 2 that when the anomalies are at the incipient level, the detection function will barely exceed the threshold, which leads to the correct detection rate to decrease lower than expected. In such cases, the anomalies exist in the measurement data, but they are likely to be ignored. Due to the miss-detected faults, the unbiasedness hypothesis of the weighted fusion algorithm is destroyed and the accuracy of the fusion estimation declines.

**Figure 5:**
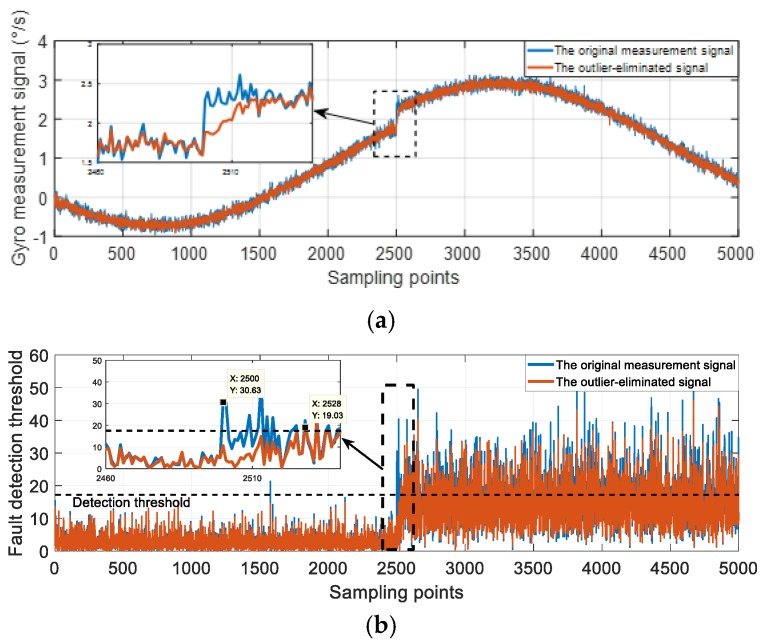
The influence of outlier eliminating. (**a**) The measurement signals; (**b**) the detection function.

On the other hand, the outlier eliminating will cause the missed detection too. There are two approaches commonly used to deal with outliers [3]: One is to improve filter functions, which alleviate the impact of outliers by setting the weighted matrix or correcting the covariance matrix at every sampling moment; another one is to replace the detected outlier points with mean values or the points from fitted curves. The outlier eliminating methods regard every anomaly point as the outliers, so the anomaly points caused by faults will also be processed by these methods. Consequently, the deviation degree of the fault will decrease. Some faults that could have been detected are missed for this reason. For example, Figure 5a shows a measurement signal with a 6σ step mutation (marked as “the original measurement signal”), and this signal is processed through the outlier eliminating method in reference [4]. The processed signal is shown as Figure 5a too. The detection functions of two signals are shown in Figure 5b. From Figure 5b, the detection function of the processed signal is lower than the original signal case at most anomaly points. The outlier eliminating will change the characteristics of the faults. To be specific, the deviation degrees of the fault will diminish, so that some faults at a low deterioration degree will be missed. Additionally, the detection function of the processed signal exceeds the threshold at the 2528-th point, which is 0.28 s later than the situation without outlier eliminating. In other words, the outlier eliminating method will cause the fault detection to be delayed.

Due to the flaws analysed above, especially the missed detection problem, the fusion estimation method cannot reach the optimal performance, because the faults and outliers break the unbiasedness of the measurement signal. To sum up, the traditional fault-tolerant strategies have some flaws, and the problems mentioned above are hard to avoid. Because of the poor stability and accuracy of MEMS sensors, these problems are more serious in MEMS-RINS.

## 5. The Fault-Tolerant Fusion Estimation

### 5.1. The Quality Index of Measurement Data 

In Section 2, the weight coefficients are calculated with the covariance matrixes, and the disadvantages have been analysed above. The fault detection function and isolation function can reflect the data anomaly in the measurement signals. So a new index is designed with these two indices.

The index $P_{i}$ to reflect the noise level is defined as:

(19) $$P_{i}=\sum_{j=1}^{n}P_{ij}$$

To avoid the excessive gap between $P_{i}$ and $F_{I}^{i}$, the first step is to standardize them as follows.

(20) $$P_{i}^{\prime }=\frac{P_{i}}{\frac{1}{n}\sum_{i=1}^{n}P_{i}}$$

(21) $$F_{I}^{i^{\prime }}=\frac{F_{I}^{i}}{\frac{1}{n}\sum_{i=1}^{n}F_{I}^{i}}$$

From the previous analysis, the larger $F_{I}^{i^{\prime }}$ and $P_{i}^{\prime }$ mean the worse quality of measurement data. In the normal situation, only using $P_{i}^{\prime }$ to calculate the weight coefficients can obtain the optimal fusion estimation. However, in the anomaly situation, $F_{I}^{i^{\prime }}$ can reflect the data quality more accurately. So the improved index is defined as follows.

(22) $$H_{i}=k_{i}F_{I}^{i^{\prime }}+(1-k_{i})P_{i}^{\prime }$$

In Equation (22), $k_{i}$ measures the extent to which the *i*-th gyro is close to being diagnosed as faulty. The value of $k_{i}$ is decided by two elements: *F_D_* and the corresponding $F_{I}^{i}$. The membership vector is defined as $a=(a_{1},a_{2})$, the calculation method of $a_{1}$ and $a_{2}$ are given by Equation (23) and Equation (24), respectively.

(23) $$a_{1}=\underset{\sim }{A}(F_{D})=\{\begin{array}{cc} 0 & F_{D}\leq T_{D1}\\ 1-e^{-{\left(\frac{F_{D}-T_{D1}T_{D}}{T_{D1}T_{D}}\right)}^{2}} & T_{D1}<F_{D} \end{array}$$

(24) $$a_{2}=\underset{\sim }{A}(F_{I}^{i^{\prime }})=\{\begin{array}{cc} 0 & F_{I}^{i^{\prime }}\leq T_{I1}\\ 1-e^{-\left(\frac{F_{I}^{i^{\prime }}-T_{I1}}{T_{I1}}\right)} & T_{I1}<F_{I}^{i^{\prime }} \end{array}$$

In Equation (23), $T_{D}$ refers to the detection threshold. $T_{D1}$ can take the value between 0 and 1 according to specific needs. Generally, when $T_{D1}$ takes 0.3, the performance is optimal. The $T_{D2}$ takes one. The $a_{1}$ is to measure the deterioration degree of the whole system. For example, $T_{D1}=0.3$ means that the system is regarded as normal when the detection function is lower than $0.3T_{D}$; when the detection function is between $0.3T_{D}$ and $T_{D}$, it can be seen as there is a minor deviation in the system measurement data, but it has not yet reached the level of being diagnosed as faults; and the higher value of detection function makes $\underset{\sim }{A}(F_{D})$ closer to one.

In Equation (24), the $T_{I1}$ takes one, which measures the extent to which the value of the isolation function of i-th gyro exceeds other gyros. When the fault detection function value is definitely high and the i-th gyro significantly exceeds the others, $\underset{\sim }{A}(F_{D})$ and $\underset{\sim }{A}(F_{I}^{i})$ are both close to one, and the i-th gyro can be considered to have faults at the time. The calculation equation of $k_{i}$ is given by Equation (25).

(25) $$k_{i}=\frac{2[\underset{\sim }{A}(F_{D})\wedge \underset{\sim }{A}(F_{I}^{i})]}{\underset{\sim }{A}(F_{D})+\underset{\sim }{A}(F_{I}^{i})}$$

### 5.2. The Weight Coefficients and Fusion Algorithm

**Figure 6:**
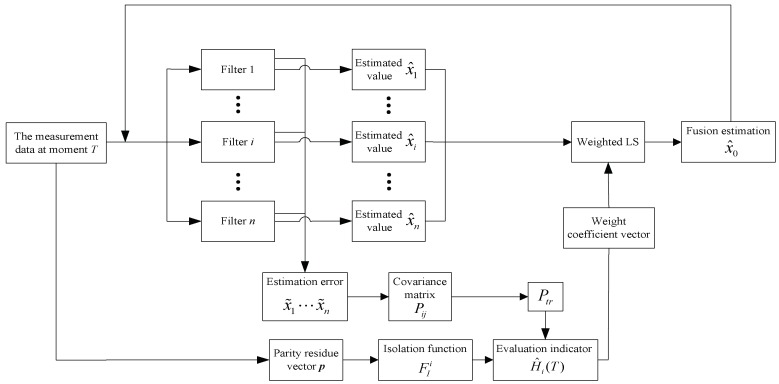
The process of the fault-tolerant fusion estimation method.

The fault-tolerant fusion estimation method adopts the framework of the distributed Kalman filtering. The algorithm structure is described in Figure 6.

After obtaining the measurement data at the *T* moment, on the one hand, the measurement data of each single gyro is sent to the corresponding local filter; on the other hand, $F_{D}$ and $F_{I}^{i}$ are calculated through the method improved in Section 3. Then from each local filter, the estimation value ${\hat{x}}_{i}$ and estimation error ${\tilde{x}}_{i}$ are calculated. After that, the covariance matrixes are calculated with ${\tilde{x}}_{i}(T)$ and ${\tilde{x}}_{j}(T)$. In practice, the error series can be regarded as uncorrelated, which can assume $P_{ij}=0(i\neq j)$. Equation (19) can also be simplified as

(26) $$P_{i}=P_{ii}$$

Next, the weight coefficient of each gyro can be calculated with *F_D_*, $F_{I}^{i}$, and $P_{tr}$ by Equations (20)–(25). Finally, the fusion estimation value can be calculated as follows.

(27) $${\hat{x}}_{0}={\left(H^{T}WH\right)}^{-1}H^{T}W\hat{X}$$

In (27), the estimated value vector $\hat{X}$ and the weight diagonal matrix W are given as follows.

(28) $$\hat{X}={\left[{\hat{x}}_{1},{\hat{x}}_{2},\cdots ,{\hat{x}}_{n}\right]}^{T}$$

(29) $$W=diag([w_{1},w_{2},\cdots w_{n}])$$

(30) $$w_{i}=\frac{1}{H_{i}\sum_{j=1}^{n}\frac{1}{H_{j}}}$$

## 6. Simulation Experiment

In order to verify the effectiveness of the proposed algorithm, the simulation experiments are conducted in this section. The dynamic gyro signals are generated from Simulink, and the different types of anomalies are injected into the signals. Several commonly used fusion methods are used as the comparison.

### 6.1. The Conditions and Signals of Experiments

**Figure 7:**
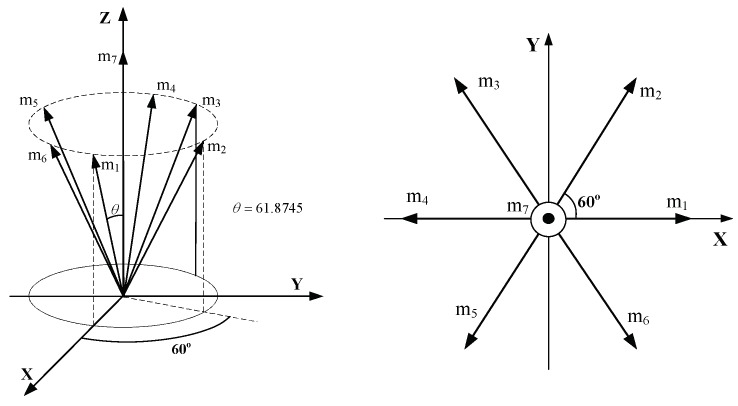
The redundant configuration of seven gyros.

To detect two anomalies at the same time, the redundant inertial measurement units (RIMU) must used more than six gyros. Besides, when the eigenvalues of ***H**^T^**H*** are equal to n/3, the fusion estimation signals have the minimum estimation error. Therefore, we choose to use seven gyros with the structure in reference [6] (shown in Figure 7), which meets $H^{T}H=7/3I_{3}$. The geometric configuration matrix of this structure is given by Equation (30).

(31) $$H={\left[\begin{array}{cccc} \begin{array}{cccc} \begin{array}{c} 0.8819\\ 0\\ 0.4714 \end{array} & \begin{array}{c} 0.4410\\ 0.7638\\ 0.4714 \end{array} & \begin{array}{c} -0.4410\\ 0.7638\\ 0.4714 \end{array} & \begin{array}{c} -0.8819\\ 0\\ 0.4714 \end{array} \end{array} & \begin{array}{c} -0.4410\\ -0.7638\\ 0.4714 \end{array} & \begin{array}{c} 0.4410\\ -0.7638\\ 0.4714 \end{array} & \begin{array}{c} 0\\ 0\\ 1 \end{array} \end{array}\right]}^{T}$$

**Figure 8:**
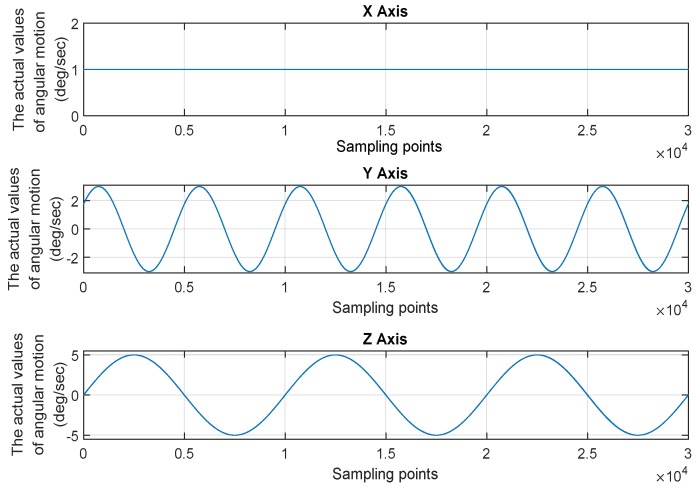
The motion mode of the aircraft.

**Figure 9:**
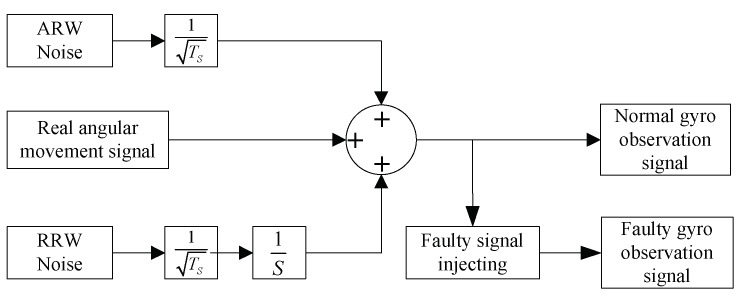
The signal generator of a single gyro.

**Figure 10:**
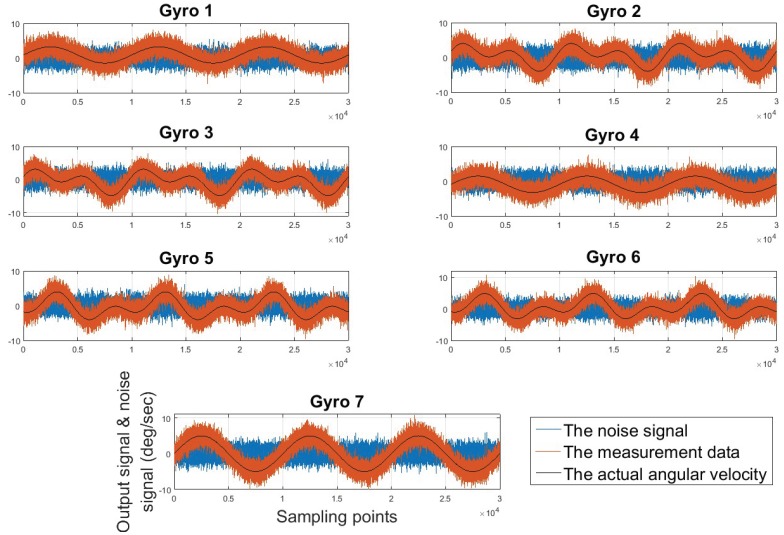
The output signals and noise.

The motion mode of the vehicle is shown in Figure 8. The sampling time is 300 s and the sampling frequency is 100 Hz. The real angular velocity measured by each gyro is calculated by Equation (8). The gyro dynamic signal generator is built by Simulink (as shown in Figure 9). Only the main random noises are considered here, which are comprised of the angular random walk (ARW) and rate random walk (RRW), and the determinate error or other random noise is not considered. The gyro output signals and noise signals are shown in Figure 10. In the figure, the Y-axis indicates the values of the noise signals and the output signals of the gyros, which the unit is deg/sec; and the X-axis indicates the sampling points. Due to the limitation of the figure size, the information of the coordinate axis is only indicated in the graph of gyro seven.

### 6.2. The Experiments and Analysis

#### 6.2.1. Single Anomaly

**Figure 11:**
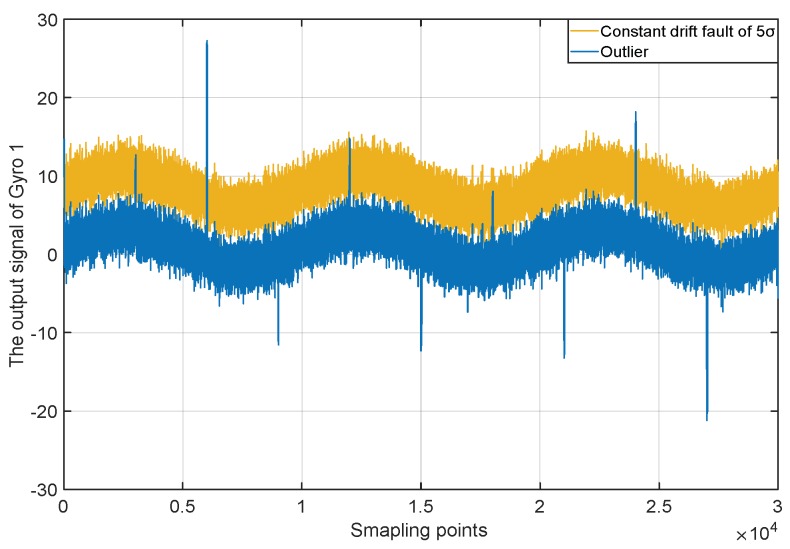
Two kinds of anomaly signals.

**Figure 12:**
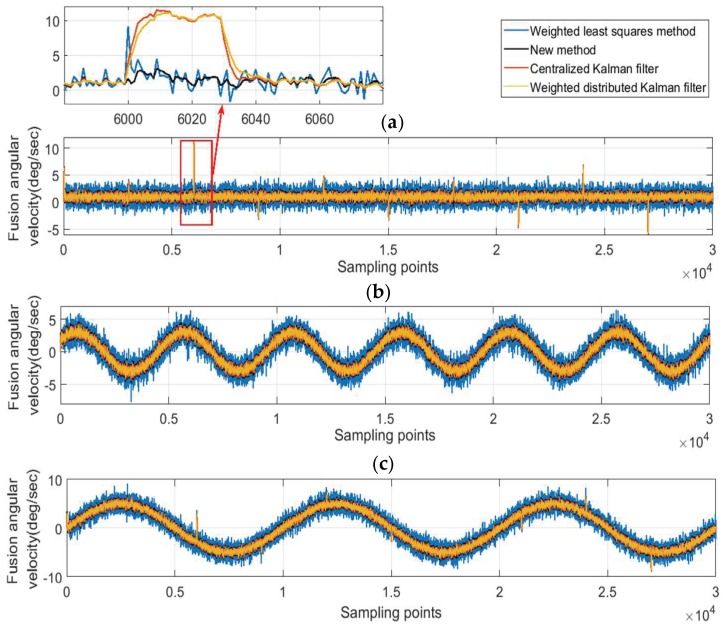
The fusion results for the case of outliers. (**a**) X Axis; (**b**) Y Axis; (**c**) Z Axis.

**Figure 13:**
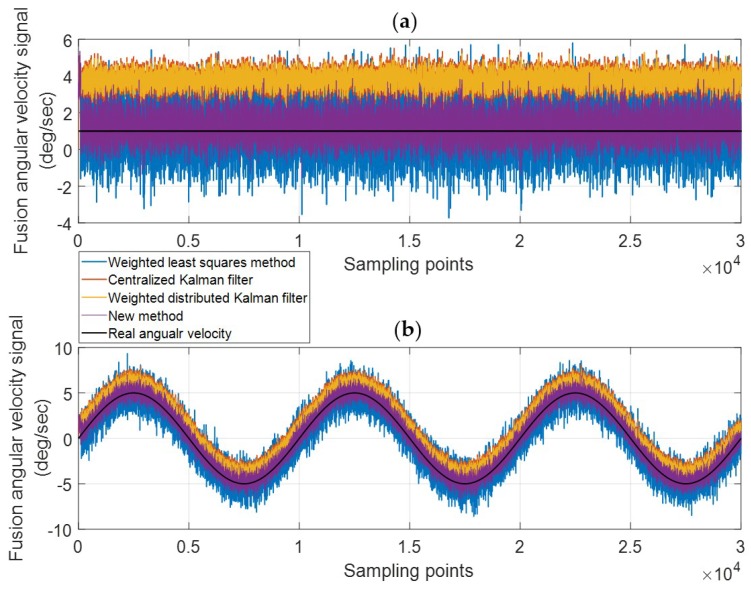
The fusion results for the constant drift case. (**a**) X Axis; (**b**) Z Axis.

To test the performance of the new method for the case of single anomaly, the outliers and the constant drift fault are injected into the normal measurement signal of gyro one in turn. The outliers are mutation anomalies that only exist a short duration. Injecting outliers is to verify the ability of tracking the quality changes of measurement data. The constant drift faults belong to the sustained anomalies, and the deviation caused by the constant drift is stable. Injecting the constant drift is to verify the ability of the correcting deviation. In the experiment, the deviation of the injected constant drift fault is 5σ. Two kinds of anomaly signals are shown in Figure 11. The weighted LS, centralized Kalman filtering, weighted distributed Kalman filtering and the new method is used to produce the fusion signals, and the fusion results are shown in Figure 12 and Figure 13.

Figure 12 shows the fusion results with outliers. Figure 12b corresponds to the Y-axis. From the first row of Equation (30), gyro one is not sensitive to the angular velocity of Y direction, so the fused signal of the Y-axis is not affected by outliers of gyro one. Figure 12b can be used as an example in the normal situation to compare the performance of these four algorithms. The detailed information of the fusion signals in Y-axis is exhibited in Table 3. Analyzing Figure 12b and Table 3, these four algorithms can reduce the noise in varying degrees: The weighted distributed Kalman filtering has the best performance; the centralized Kalman filtering and the new method are less effective, but their performances are roughly equivalent to the weighted distributed Kalman filtering; the weighted LS has the worst performance.

Figure 12a,c corresponds to the X-axis and the Z-axis, respectively. It can be seen that four methods can reduce the deviation of the outliers to different extents. The new method has the best eliminating effect of outliers, followed by the weighted LS; the centralized Kalman filtering and weighted distributed Kalman filtering perform poorly. The weight coefficients of the weighted LS are calculated based on the innovation of the previous moments. If the gyro works normally at k−1 moment, the innovation is also in a normal range. Then the outlier appears at k moment, but its weight coefficient is still calculated based on the normal innovation of the k−1 moment. So the weight coefficients of the weighted LS have a one-step delay compared to the data quality. Therefore, there is still a huge deviation in the fusion data at the moment when the outliers first appear.

Figure 13 shows the fusion results with the constant drift fault. From the analysis above, the fusion signal of the Y-axis is not affected by gyro one, so only the fusion results of the X-axis and the Z-axis are shown in the figure. The constant drift value in the experiment is $5\sigma \approx 7.495\;{(}^{\circ }/s)$. 

As Figure 13 shows, four methods can correct the deviation of the gyro one in varying degrees. The details of the fusion signal error of the X-axis and Z-axis is shown in Table 4. The analysis shows that the centralized Kalman filtering and the weighted distributed Kalman filtering can control the variance to a small level, but these two methods have a poor performance in correcting the constant drift; the weighted LS method can handle the bias better, but the noise is still large after the fusion; the new method performs best and has the smallest estimation error.

#### 6.2.2. Double Anomalies

In the situation of double anomalies, the anomaly signals are injected simultaneously into gyro one and gyro two, and the injecting scheme is shown in Table 5.

The gyro measurement signals with anomalies are similar to Figure 11, so they are not shown again here. Additionally, the signal of the gyro one does not affect the fusion signal of the Y-axis. For the Y-axis, the anomalies appearing simultaneously in the gyro one and gyro two are only equivalent to a single anomaly of gyro two, and the single anomaly situation is not discussed here again.

**Figure 14:**
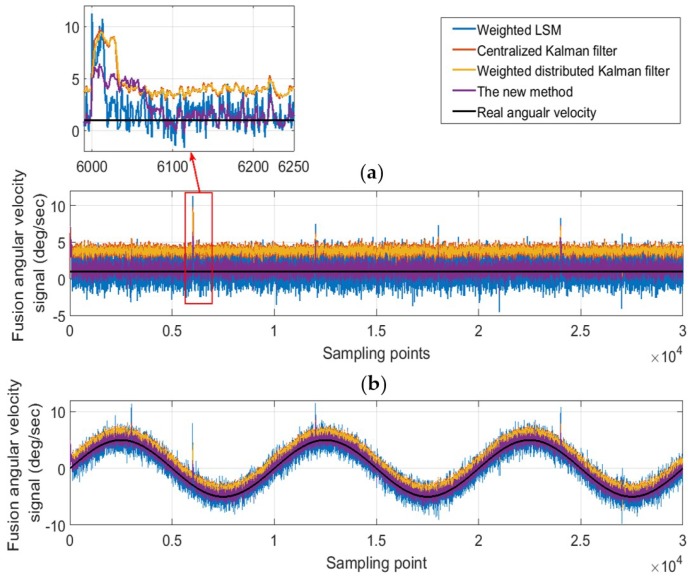
The fusion results for the case of outliers and the constant drift fault. (**a**) X Axis; (**b**) Z Axis.

Figure 14 shows the situation that the constant drift fault and outliers occurs simultaneously and the details are shown in Table 3. When signal of gyro two has no outlier, the fusion result is consistent with the single-anomaly situation. From Figure 14 and Table 6, when outliers exit in signal of gyro two, the method proposed in this paper is obviously better than the other three methods, but the effect is slightly worse than the single-anomaly situation.

**Figure 15:**
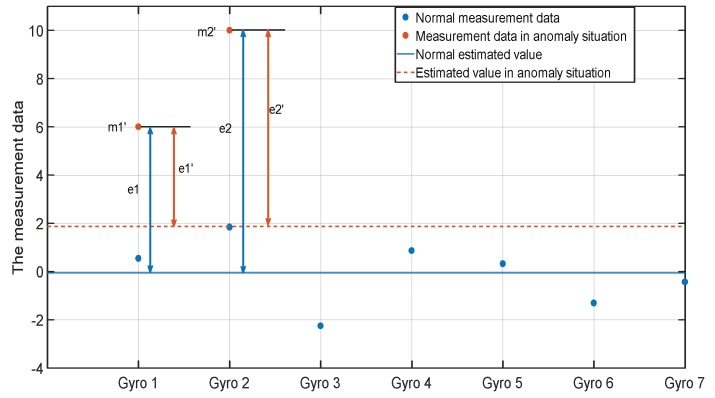
An example of anomalies in the same direction.

When the two anomalies are in the same direction, that is, the errors caused by the anomalies are both positive or both negative, the performance of the various methods will degrade; when the two anomalies are reversed, the performance is similar with the single-anomaly situation. For example, as shown in Figure 15, when the same direction anomalies occur in gyro one and gyro two, the estimated value will drift in the anomaly direction. As a result, the innovation will decrease, which will cause the weight coefficients of the anomaly sensors to get larger, and thus the performance is supposed to degrade. Nevertheless, such performance degradation is acceptable and has little impact on the overall system.

**Figure 16:**
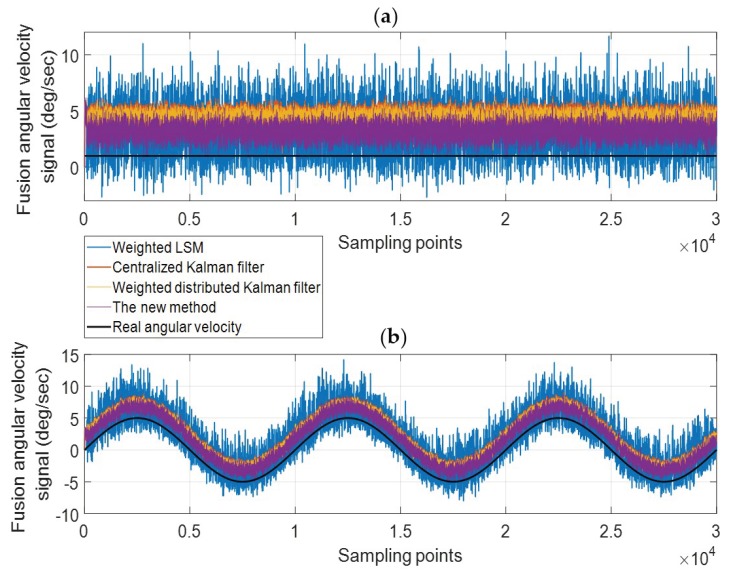
The fusion results for the case of two constant drift faults. (**a**) X Axis; (**b**) Z Axis.

Figure 16 shows the fusion results of two simultaneous constant drift faults. Table 7 shows the details of the fusion signal error. Similar to the case of single anomaly, the centralized Kalman filtering and weighted distributed Kalman filtering produce the signals with small noise, but the drift degree is much higher than the other two methods; the noise and drift degree of weighted LS are larger than the new method. In addition, the signal noise obtained by the centralized Kalman filtering and the weighted distributed Kalman filtering have a small increase compared to the single-anomaly case. It proves, on the other hand, that these two methods only consider the impact of the noise. It only can be used when the observation data is unbiased.

#### 6.2.3. The Simulation Experiments through Different Configurations

In the sections above, the proposed method is compared with three widely-used methods, and the advantages of the new method are verified. In this section, the performance of the new method is tested based on different configurations, which are given in Table 8.

In this set of experiments, the conditions and original signal generators are set the same as above, and a 5σ constant drift fault is injected into the first gyro of each configuration. Analysing Table 8, elements (2,1) and (3,1) take 0 in some configurations, which means that, in such configurations, the measurement data of gyro one is unrelated to the fusion estimation signals in the Y-axis or Z-axis. For this reason, the comparison only considers the output signals in the X-axis. Table 9 exhibits the average estimation error of the fusion estimation signals in the X-axis from four methods adopted above, the unit of the number in Table 9 is °/s.

In configuration 1, 2, 5 and 7, the error of four methods did not differ from each other very much. These configurations only have two gyros whose projections on the X-axis are not equal to 0. In other words, in these configurations, signals in the X-axis only depend on two gyros. If the fault appears in one of the two gyros, the faulty sensor is hard to isolate correctly. Further, in these configurations, the error is inverse proportional to the projection value. The relation between the configuration and measurement accuracy can be analysed through geometric dilution of precision (GDOP) and its related theory. This paper mainly focuses on the method, so no further research would be conducted here. To compare the four methods, in configuration 1, 2, 5 and 7, the weighted LS performs worst, and rest three methods have a consistent performance; in configuration 3, 4, 6, 8 and 9, the new method performs much better than other three methods. Configuration nine is the most typical example. In configuration nine, the error of the new method is about half of the weighted least squares, 15.51% of the centralized Kalman filter, and 15.52% of the weighted distributed Kalman filtering. In general, the new method has an improved ability to eliminate the influence of faults.

## 7. Conclusions

The fault-tolerant fusion is a necessary part in MEMS RINS. But most methods are built on the unbiasedness hypothesis. Considering the problems of outliers and the instability of MEMS IMU, the practical performance degradation is inevitable for this kind of method. To solve the problems, we use the parity vector to indicate the unbiasedness. Combining with the covariance matrix, an improved index is designed to reflect the measurement data. Further, the improved algorithm is proposed. The performance is tested through a series of simulation experiments. The simulation experiments show that this new method can reduce the deviation caused by the abnormal data well and control the noise to a low level. In the cases of the single anomaly and double anomalies, the developed method has a better performance than the weighted LS, centralized Kalman filtering and weighted distributed Kalman filtering. Lastly, to analyse the performance further, the new method is tested through nine configurations, and the new method also achieves the smallest error level.

## Figures and Tables

**Table 1:** The detailed information of the fused signals.

The Fusion Signals in Different Situations	Noise Variance of the Fusion Signal	Constant Drift of the Fusion Signal
The Normal Case (°/s)	7.053 × 10^−4^	2.508 × 10^−6^
The Faulty Case (°/s)	8.592 × 10^−4^	1.250

**Table 2:** The performance test with different anomalies.

Deviation Degree	1*σ*	2*σ*	3σ	4σ	5σ	6*σ*
The frequency at which the detection function exceeds the threshold when an exception occurs	0.0014	0.0061	0.1326	0.3128	0.5944	0.7587

**Table 3:** The average estimation error of different fusion method in the Y-axis.

	Weighted Least Squares (LS)	Centralized Kalman Filtering	Weighted Distributed Kalman Filtering	The New Method
Average estimation error (°/s)	1.0179	0.4312	0.3508	0.4662

**Table 4:** The details of fusion signal error for the constant drift fault case.

		Weighted LS	Centralized Kalman Filtering	Weighted Distributed Kalman Filtering	The New Method
X Axis	Constant drift (°/s)	0.3102	2.8081	2.8052	0.2032
	Noise variance ((°/s)^2^)	1.5383	0.1865	0.1307	0.4858
	Average estimation error (°/s)	1.0236	2.8082	2.8054	0.5588
Z Axis	Constant drift (°/s)	0.1815	1.5089	1.5074	0.1165
	Noise variance ((°/s)^2^)	1.1820	0.1892	0.1255	0.3271
	Average estimation error (°/s)	0.8782	1.5091	1.5075	0.4570

**Table 5:** The scheme of anomaly injecting.

	Gyro 1	Gyro 2
1	Constant drift fault of 5σ	Outliers
2	Constant drift fault of 5σ	Constant drift fault of 3σ

**Table 6:** The details of fusion signal error for the outlier case.

		Weighted LS	Centralized Kalman Filtering	Weighted Distributed Kalman Filtering	The New Method
Average estimation error (°/s)	X axis	1.0388	2. 8126	1.5183	0.5834
	Z axis	0.8906	1.5201	1.5183	0.4747

**Table 7:** The details of the fusion signal error.

		Weighted LS	Centralized Kalman Filtering	Weighted Distributed Kalman Filtering	The New Method
X Axis	Constant drift (°/s)	2.4942	3.6580	3.6541	2.1593
	Noise variance ((°/s)^2^)	3.2346	0.1867	0.1337	0.4463
	Average estimation error (°/s)	2.6148	3.6580	3.6543	2.1597
Z Axis	Constant drift (°/s)	1.8813	2.4173	2.4151	1.7874
	Noise variance ((°/s)^2^)	2.8795	0.1856	0.1280	0.3641
	Average estimation error (°/s)	2.0488	2.4174	2.4151	1.7878

**Table 8:** Different rotation inertial navigation system (RINS) configurations adopted in experiments.

Configuration 1 (4 gyros)	Configuration 2 (4 gyros)	Configuration 3 (5 gyros)
$H=\left[\begin{array}{ccc} \begin{array}{c} 1\\ 0\\ 0\\ 0.5774 \end{array} & \begin{array}{c} 0\\ 1\\ 0\\ 0.5774 \end{array} & \begin{array}{c} 0\\ 0\\ 1\\ 0.5774 \end{array} \end{array}\right]$	$H=\left[\begin{array}{ccc} \begin{array}{c} 0.8164\\ 0\\ -0.8164\\ 0 \end{array} & \begin{array}{c} 0\\ 0.8164\\ 0\\ -0.8164 \end{array} & \begin{array}{c} 0.5774\\ 0.5774\\ 0.5774\\ 0.5774 \end{array} \end{array}\right]$	$H=\left[\begin{array}{ccc} \begin{array}{c} \begin{array}{c} 1\\ 0 \end{array}\\ 0.4714\\ -0.6440\\ 0.1725 \end{array} & \begin{array}{c} \begin{array}{c} 0\\ 1 \end{array}\\ 0.4714\\ 0.1725\\ -0.6440 \end{array} & \begin{array}{c} \begin{array}{c} 0\\ 0 \end{array}\\ 0.7454\\ 0.7454\\ 0.7454 \end{array} \end{array}\right]$
Configuration 4 (5 gyros)	Configuration 5 (6 gyros)	Configuration 6 (6 gyros)
$H=\left[\begin{array}{ccc} \begin{array}{c} \begin{array}{c} 1\\ 0 \end{array}\\ 0\\ 0.5774\\ 0.5774 \end{array} & \begin{array}{c} \begin{array}{c} 0\\ 1 \end{array}\\ 0\\ 0.5774\\ 0.5774 \end{array} & \begin{array}{c} \begin{array}{c} 0\\ 0 \end{array}\\ 1\\ 0.5774\\ 0.5774 \end{array} \end{array}\right]$	$H=\left[\begin{array}{ccc} \begin{array}{c} \begin{array}{c} 1\\ 1\\ 0 \end{array}\\ 0\\ 0\\ 0 \end{array} & \begin{array}{c} \begin{array}{c} 0\\ 0\\ 1 \end{array}\\ 1\\ 0\\ 0 \end{array} & \begin{array}{c} \begin{array}{c} 0\\ 0\\ 0 \end{array}\\ 0\\ 1\\ 1 \end{array} \end{array}\right]$	$H=\left[\begin{array}{ccc} \begin{array}{c} \begin{array}{c} 0.5257\\ -0.5257\\ 0.8507 \end{array}\\ 0.8507\\ 0\\ 0 \end{array} & \begin{array}{c} \begin{array}{c} 0\\ 0\\ 0.5257 \end{array}\\ -0.5257\\ 0.8507\\ 0.8507 \end{array} & \begin{array}{c} \begin{array}{c} 0.8507\\ 0.8507\\ 0 \end{array}\\ 0\\ 0.5257\\ -0.5257 \end{array} \end{array}\right]$
Configuration 7 (7 gyros)	Configuration 8 (7 gyros)	Configuration 9 (7 gyros)
$H=\left[\begin{array}{ccc} \begin{array}{c} 1\\ 1\\ 0\\ \begin{array}{c} 0\\ 0\\ 0\\ 0 \end{array} \end{array} & \begin{array}{c} 0\\ 0\\ 1\\ \begin{array}{c} 1\\ 0\\ 0\\ 0 \end{array} \end{array} & \begin{array}{c} 0\\ 0\\ 0\\ \begin{array}{c} 0\\ 1\\ 1\\ 1 \end{array} \end{array} \end{array}\right]$	$H=\left[\begin{array}{ccc} \begin{array}{c} 0.8165\\ 0.5091\\ -0.1817\\ \begin{array}{c} -0.7356\\ -0.7356\\ -0.1817\\ 0.5091 \end{array} \end{array} & \begin{array}{c} 0\\ 0.6384\\ 0.7960\\ \begin{array}{c} 0.3543\\ -0.3543\\ -0.7960\\ -0.6384 \end{array} \end{array} & \begin{array}{c} 0.5774\\ 0.5774\\ 0.5774\\ \begin{array}{c} 0.5774\\ 0.5774\\ 0.5774\\ 0.5774 \end{array} \end{array} \end{array}\right]$	$H=\left[\begin{array}{c} \begin{array}{ccc} 0.8819 & 0 & 0.4714\\ 0.4410 & \;0.7638\; & 0.4714\\ -0.4410 & \;0.7638\; & 0.4714 \end{array}\\ \begin{array}{ccc} -0.8819 & 0 & 0.4714\\ -0.4410 & -0.7638 & 0.4714\\ 0.4410 & -0.7638 & 0.4714\\ 0 & 0 & 1 \end{array} \end{array}\right]$

**Table 9:** The performance comparison among different configurations.

	1	2	3	4	5	6	7	8	9
Weighted LS	0.5489	0.3778	0.2468	0.5124	0.3146	0.0703	0.3681	0.0710	0.0691
Centralized Kalman filtering	0.5001	0.3676	0.3598	0.4666	0.3004	0.1572	0.3003	0.2101	0.2263
Weighted distributed Kalman filtering	0.5002	0.3674	0.3600	0.4668	0.3004	0.1572	0.3001	0.2100	0.2261
The new method	0.5000	0.3674	0.2723	0.4617	0.3004	0.0359	0.3000	0.0341	0.0351
